# The intricate interplay of microbial metabolomics and carcinogenesis: a spotlight on mechanistic pathways, clinical implications and methodological challenges

**DOI:** 10.3389/fcimb.2026.1787954

**Published:** 2026-05-29

**Authors:** Mostafa Eysha, Malak Fouani, Malak Munir, Mariah Black, Anna Long, Mohanad Elchouemi, Sharda Singh, Muhammad Bilal Abid

**Affiliations:** 1 Department of Internal Medicine, Paul L. Foster School of Medicine, Texas Tech University Health Sciences Center El Paso, El Paso, TX, United States; 2 Department of Neurology, Duke University Medical Center, Durham, NC, United States; 3 Department of Medicine, Cedars-Sinai Medical Center, Los Angeles, CA, United States; 4 Department of Surgery, Duke University School of Medicine, Durham, NC, United States; 5 Department of Hematology/Oncology, UMC Cancer Center, Texas Tech University Health Sciences Center, Lubbock, TX, United States

**Keywords:** biomarkers, cancer, carcinogenesis, immunotherapy, mass spectrometry, metabolomics, microbial metabolites, nuclear magnetic resonance spectroscopy

## Abstract

Cancer is increasingly recognized to involve profound metabolic reprogramming, where the resident human microbiome acts as a “second genome” that fundamentally influences health and disease. At the intersection of oncology and microbiology lies the microbial metabolome, a comprehensive set of small-molecule metabolites that serve as the primary functional effectors between the microbiome and the host. We synthesize mechanistic evidence across hematologic malignancies as well as solid tumors including colorectal, pancreatic, breast, liver, and head and neck cancers. Pro-carcinogenic metabolites, such as secondary bile acids and bacterial genotoxins like colibactin, drive malignancy through chronic inflammation, direct DNA damage, and oncogenic signaling. Conversely, protective metabolites, predominantly short-chain fatty acids like butyrate, counteract cancer progression through immune modulation, selective apoptosis, and epigenetic regulation. This review examines the microbial metabolome as a “double-edged sword” in carcinogenesis, detailing how these molecules can either promote or suppress tumorigenesis depending on their identity, concentration, and the host environment. The review further explores the translational potential of microbial metabolomics in clinical oncology. We highlight the emerging role of metabolites as diagnostic and prognostic biomarkers and their capacity to modulate the efficacy and toxicity of chemotherapy and immunotherapy. Finally, we address critical methodological hurdles, including the need for standardization and established causality while providing a roadmap for integrating metabolomic profiling into a new era of personalized precision oncology.

## 1 Introduction

**Figure 1:**
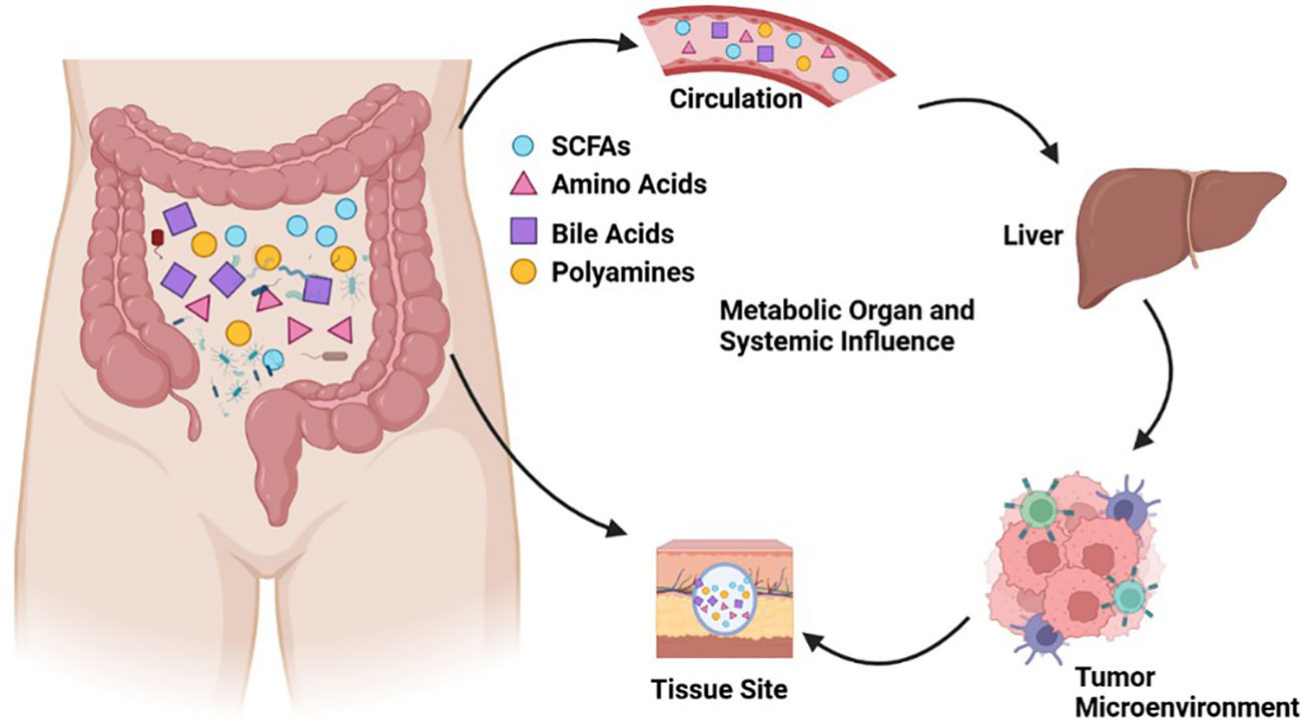
Gut and tissue-associated microbiome as a metabolic organ with systemic influence. The gut and tissue-associated microbiome functions as a metabolic organ that produces bioactive metabolites, including short-chain fatty acids (SCFAs), amino acids, bile acids, and polyamines. These metabolites enter the systemic circulation, are processed by the liver, and subsequently influence the tumor microenvironment. Circulating metabolites also reach peripheral tissues, where similar microbial-derived metabolites are present locally. Together, this bidirectional pathway highlights the systemic metabolic influence of the microbiome across organs and tissues.

Cancer involves profound metabolic reprogramming that drives aggressive cell proliferation, immune evasion, and metastasis (**Figure 1****) (**Zhu, 2024). The interplay between the human host and resident microbial communities has emerged as a frontier in medical science, fundamentally reshaping our understanding of health and disease. At the intersection of microbiology, immunology, and oncology lies the microbial metabolome, the comprehensive study of small-molecule metabolites that mediate microbiome-host interactions (Perez Escriva et al., 2025). This interaction is proposed to play an essential role in inflammation, DNA integrity, metabolism, and immune surveillance within the tumor microenvironment (TME).

### 1.1 Defining the human microbiome and metabolomics

#### 1.1.1 The human microbiome: a “second genome”

The human microbiome comprises the vast communities of bacteria, archaea, fungi, and viruses inhabiting nearly every barrier surface of the human body (Aggarwal et al., 2023). This microbial ecosystem is estimated to be on the same order of magnitude as human cells, and its collective genetic content, the “metagenome,” contains an estimated 50 to 100 times more genes than the human genome itself, leading to the concept of the microbiome as a “second genome” (Ogunrinola et al., 2020). The biochemical independence of microbial carcinogenic pathways from the host genome has been directly demonstrated: six enzymes encoded within the bacterial *bai* operon are necessary and sufficient for the full conversion of primary to secondary bile acids (BA), a capacity entirely absents from the human genome (Funabashi et al., 2020). More recent studies highlighted the microbiome’s pivotal role in cancer progression, acting either through direct host-cell interactions or by releasing bioactive compounds such as microbial metabolites (Abid, 2019). We will discuss this in detail throughout the review.

#### 1.1.2 Metabolomics

Metabolomics systematically characterizes the metabolome, defined as the complete set of small-molecule metabolites within a biological system (Chen et al., 2022). Because metabolites are the end products of cellular processes, the metabolome provides an instantaneous snapshot of an organism’s physiology, representing the ultimate, integrated readout of its genetic and environmental interactions. Metabolites, in addition to being produced directly by the host, can be derived from the host microbiota. Rapid advancements in analytical platforms such as liquid chromatography-mass spectrometry (LC-MS) and nuclear magnetic resonance (NMR) spectroscopy enabled high-throughput analysis (Chen et al., 2022). Despite this technological evolution, metabolomics has lagged behind genomics and transcriptomics in clinical applications, particularly in oncology (Kohli and Poulogiannis, 2025). A primary reason for this deficiency is the fundamental complexity: the metabolome of a biological sample is not only the product of the host but also a combination of host and microbial activities (Johnson et al., 2016). Historically, cancer research has focused on altered host cell metabolism, and consequently, metabolomic studies have predominantly characterized the host metabolome. The vast contribution of the microbial metabolome remains a comparatively under-researched frontier (Huang and Mao, 2022). To differentiate between the host and the microbial, this requires specialized experimental systems like germ-free animal models, antibiotic perturbation, isotope tracing, or integrated metagenomic and metabolomic analyses. Among germ-free models, the Oligo-Mouse-Microbiota 12 (OMM-12) consortium, a defined community of 12 fully genome-sequenced bacterial strains, has emerged as a particularly tractable platform for dissecting the contributions of microbial metabolites to carcinogenesis. Originally developed by Brugiroux et al., the OMM-12 model has colonized mice under germ-free conditions stably across generations and has been validated for reproducibility across independent facilities (Brugiroux et al., 2016; Eberl et al., 2020). This has enabled mechanistic attribution of specific metabolic outputs, such as secondary bile acid (SBA) production and short-chain fatty acid (SCFA) fermentation, to defined bacterial gene functions rather than to complex, undefined communities. Complementing these *in vivo* models, patient-derived organoid (PDO) co-culture systems extend this mechanistic framework to human tissue. PDOs can be co-exposed to defined microbial species, conditioned metabolite media, or autologous immune cells, thereby providing a translational bridge between metagenomic observations and causal inference within a human cellular context (Pleguezuelos-Manzano et al., 2020). Together, these experimental systems address the core methodological challenge of separating host from microbial metabolome contributions, a challenge that has historically limited the field.

### 1.2 The emerging role of microbial metabolites in cancer biology

Microbial metabolites are primary effectors linking the microbiome to cancer. These molecules produced either *de novo* by microbes or via biotransformation of dietary substrates and host-derived compounds, act as potent signaling agents rather than inert byproducts (Huang and Mao, 2022). The recognition of metabolites as the connection between the microbiome and the cancer represents a paradigm shift in the field, moving from a purely compositional analysis of which microbes are present to a functional analysis of what those microbes are doing (**Figure 1**) (Yang et al., 2023). Functionally similar metabolic outputs can arise from different microbial communities, explaining difficulties in defining a universal “cancer microbiome” taxonomically (Zhou et al., 2024a).

### 1.3 Objectives and scope of the review

This review aims to comprehensively examine the microbial metabolome in cancer, emphasizing mechanistic insights and translational implications, including the dual role in carcinogenesis, clinical applications, methodological changes, and future directions. Although the human microbiome encompasses bacteria, archaea, fungi, and viruses, this review focuses on the bacterial microbiome and its metabolic products. This reflects the current state of experimental evidence as bacteria are the source of the metabolite classes with the most directly demonstrated mechanistic roles in carcinogenesis, including SBAs and genotoxins such as colibactin (Funabashi et al., 2020; Pleguezuelos-Manzano et al., 2020). Unless otherwise specified, ‘microbiome’ throughout this review refers to the bacterial microbiome.

## 2 The dual role of microbial metabolites in carcinogenesis

Anaerobic gut flora ferment dietary and host-derived substrates to produce metabolites that influence host physiology (Flint et al., 2012; Marcobal et al., 2013). These metabolites have a dual role in carcinogenesis, either by promoting or suppressing tumorigenesis depending on their identity, concentration, and the host microenvironment. Major molecular classes include SCFAs, SBAs, bacterial toxins/genotoxins, polyamines, hydrogen sulfide, protein fermentation products (e.g., indoles, phenols, p-cresol), and tryptophan-derived catabolites. The colon harbors the densest and most metabolically active microbial community in the human body, rich in *Firmicutes* and *Bacteroidetes*, in which key species such as *Akkermansia muciniphila, Bifidobacterium*, and *Faecalibacterium prausnitzii* disproportionately affect metabolite production, epithelial barrier integrity, and therapeutic response (Sivan et al., 2015; Vetizou et al., 2015; Routy et al., 2018; Tanoue et al., 2019; Ocariz-Diez et al., 2020). The mechanistic evidence provided in this section derives primarily from preclinical models of gastrointestinal malignancies, including *in vitro* studies using human cancer cell lines, murine models, and human intestinal organoids, with validation from human tumor specimens where available. Additionally, it is important to note that metabolite effects may be context dependent, varying by concentration, tissue microenvironment, and cancer type.

### 2.1 Pro-carcinogenic mechanisms

**Figure 2:**
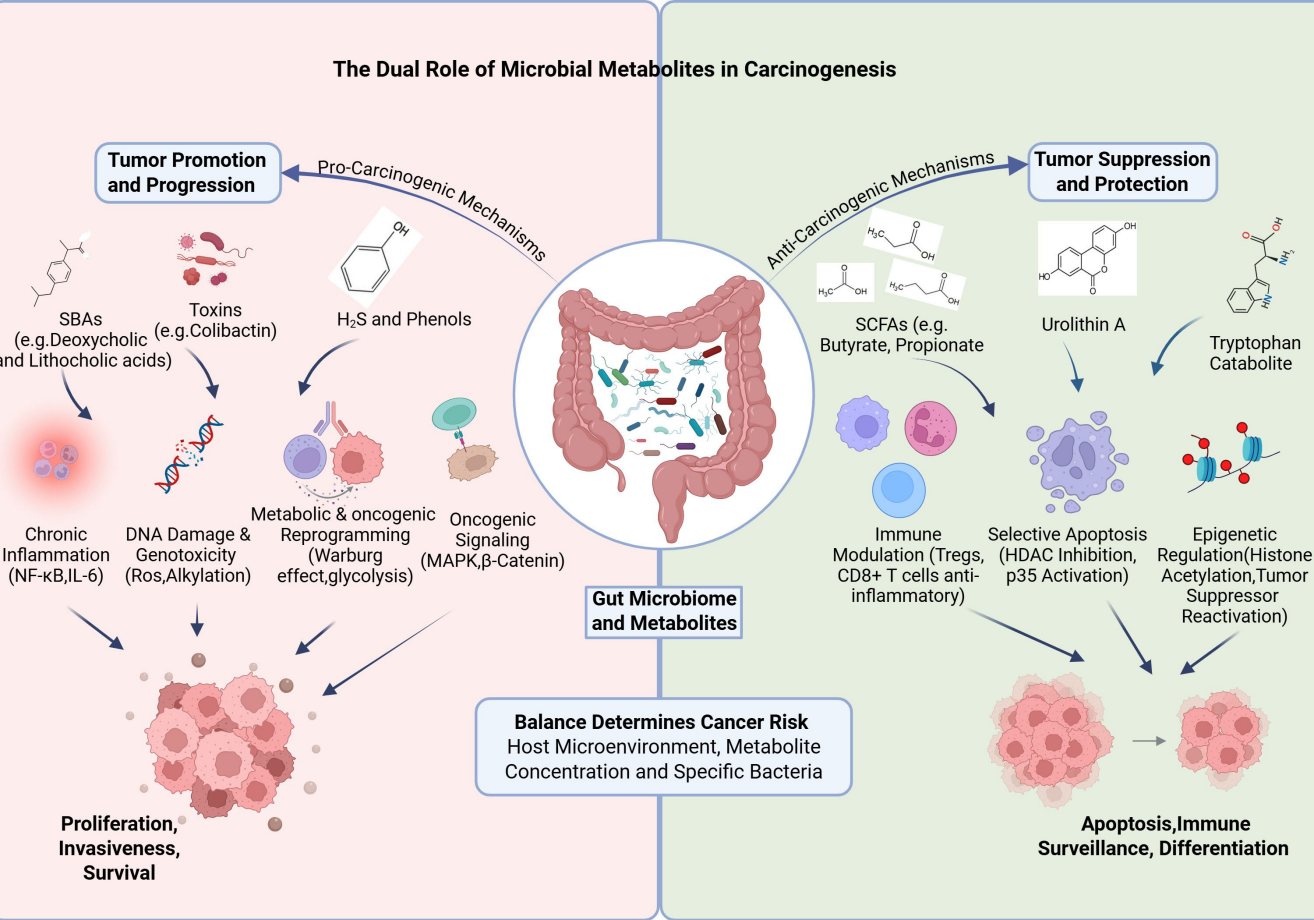
The dual role of microbial metabolites in carcinogenesis. Microbial metabolites derived from the large intestine exert opposing effects on cancer development. Pro-carcinogenic mechanisms involve metabolites such as secondary bile acids (SBAs), toxins, hydrogen sulfide (H_2_S), and phenols, which promote tumor progression through chronic inflammation (NF-κB, IL-6), DNA damage and genotoxicity (ROS, alkylation), metabolic reprogramming (Warburg effect, glycolysis), and activation of oncogenic signaling pathways (MAPK, β-catenin), ultimately leading to increased cancer proliferation and invasiveness. In contrast, anti-carcinogenic mechanisms are mediated by metabolites including short-chain fatty acids (SCFAs; e.g., butyrate and propionate), urolithin A, and tryptophan catabolites, which promote tumor suppression via immune modulation (Tregs, CD8^+^ T cells, anti-inflammatory effects), selective apoptosis (HDAC inhibition, p53 activation), and epigenetic regulation (histone acetylation, tumor suppressor gene reactivation), resulting in cancer cell apoptosis, immune surveillance, and differentiation. The balance between these opposing pathways—shaped by the host microenvironment, metabolite concentrations, and specific microbial communities—ultimately determines cancer risk.

Tumor-promoting metabolites drive malignancy through chronic inflammation, genotoxicity, metabolic reprogramming, and oncogenic signaling. SBAs, particularly deoxycholic lithocholic acids, are pro-inflammatory metabolites (**Figure 2**). These hydrophobic compounds are formed endogenously through two main pathways: the action of bacterial bile salt hydrolases and 7α-dehydroxylation by specific *Clostridium* species (Ridlon et al., 2006; Jones et al., 2008; Ridlon and Hylemon, 2012). Upon interaction with the cell, these hydrophobic compounds generate reactive oxygen and nitrogen species, ultimately leading to DNA damage (Bernstein et al., 2009). Specifically, *in vitro* studies using human colon cancer cell lines have shown that deoxycholic acid (DCA) increases DNA strand breaks and mitochondrial oxidative stress while activating the mitogen-activated protein kinase (MAPK) pathways via epidermal growth factor receptor (EGFR), thereby promoting cancer proliferation and invasiveness (Im and Martinez, 2004; Jean-Louis et al., 2006). Similarly, lithocholic acid (LCA) promotes cancer invasiveness through increased expression of matrix metalloproteinases (MMP) and urokinase-type plasminogen activator in colonic carcinoma cells (Halvorsen et al., 2000; Baek et al., 2010).

Furthermore, the nuclear factor kappa B (NF-κB) pathway amplified via interleukin(IL)-6/signal transducer and activator of transcription 3 (STAT3) feedback drives inflammation-mediated tumorigenesis (Iliopoulos et al., 2009; De Simone et al., 2015; Johnson et al., 2018; Peng et al., 2020; Hirano, 2021; Kearns, 2025). Pathogenic bacteria contribute through activation of the pattern recognition toll-like receptors (TLR), with evidence from CRC cell lines of *Fusobacterium nucleatum, Peptostreptococcus anaerobius, and Enterococcus faecalis* activating TLR2/4 signaling via myeloid differentiation primary response 88 (MYD88) to drive NF-κB activation and pro-inflammatory cytokine production (Yang et al., 2017; Di Lorenzo et al., 2020; Sheikh et al., 2021; Galasso et al., 2025).

Additionally, protein digestion, specifically aromatic amino acid fermentation products such as phenylacetic acid, phenols, indoles, and p-cresol, along with hydrogen sulfide from sulfur-metabolizing bacteria (*Desulfovibrio* species), mediates inflammation (Kaur et al., 2017; Tan et al., 2023). Current evidence suggests that hydrogen sulfide exhibits concentration-dependent effects on cellular function. At low concentrations, it scavenges reactive oxygen species and provides cytoprotective benefits (Blachier et al., 2019; Blachier et al., 2021; Mallardi et al., 2025). However, as reported in rodent models, at colonic concentrations (>0.25–2 mM), sulfide becomes genotoxic, impairs mitochondrial respiration, disrupts epithelial barrier integrity, and promotes inflammation and carcinogenesis (Beaumont et al., 2016; Blachier et al., 2019; Blachier et al., 2021; Mallardi et al., 2025). Sulfide also directly promotes protein kinase B (Akt) and extracellular signal-regular kinase (ERK) phosphorylation, pathways that enhance cancer cell survival (Giuffre et al., 2020; Tao et al., 2024).

Other genotoxic metabolites, specifically bacterial toxins, promote carcinogenesis by activating oncogenic pathways. Colibactin, produced by pks+ *Escherichia coli* strains, alkylates DNA to form interstrand crosslinks, generating characteristic mutational signatures identified in human CRC specimens and further validated in organoid models, found in over 12% of CRC cases (Pleguezuelos-Manzano et al., 2020; Rosendahl Huber et al., 2024). The toxin activates the ataxia telangiectasia mutated/ataxia telangiectasia and Rad3-related checkpoint kinase 2 (ATM/ATR-Chk2) pathway, inducing DNA damage responses and p53 protein accumulation, as demonstrated across *in vitro* murine, and human biopsy studies (Cougnoux et al., 2014; Iftekhar et al., 2021; Dougherty et al., 2023). In p53-mutated CRC cases (43-60%) (Russo et al., 2005; Nakayama and Oshima, 2019; Liebl and Hofmann, 2021), colibactin instead drives tumor progression through pathways such as Wnt/β-catenin signaling (Iftekhar et al., 2021).

Various bacterial toxins also contribute to carcinogenesis, such as tilimycin from *Klebsiella oxytoca*, which induces N^2^-guanine alkylation in murine models, with the toxin detected in human colitis specimens (Unterhauser et al., 2019; Poltl et al., 2023). In contrast, cytolethal distending toxin from pathogenic *E. coli* and *Campylobacter jejuni* acts as a potent deoxyribonuclease, inducing double-strand DNA breaks, cell cycle arrest, and apoptosis (Guerra et al., 2011; Bezine et al., 2014; Lai et al., 2021). Beyond DNA damage, trans-3-indoleacrylic acid from *P. anaerobius* facilitates carcinogenesis by inhibiting ferroptosis through aldehyde dehydrogenase 1 family member A3 (ALDH1A3) upregulation in CRC cell lines, thereby creating ferroptosis-resistant phenotypes that allow damaged cells to survive (Cui et al., 2024; Zhang R. et al., 2024).

Microbial metabolites drive metabolic reprogramming that supports cancer development. For example, *F. nucleatum* produces formate, promoting the Warburg effect and integrating into one-carbon metabolism pathways essential for nucleotide synthesis (Oizel et al., 2020). At the same time, SBAs have been shown to disrupt mitochondrial membrane integrity in hepatocyte and muscle cell models, forcing glycolytic dependence and generating acidic, immunosuppressive microenvironments (Rolo et al., 2000; Sousa et al., 2015; Abrigo et al., 2023). These metabolic disruptions are amplified by pathobiont-activated oncogenic signaling, where *F. nucleatum’s* FadA adhesin binds E-cadherin to activate β-catenin signaling in CRC cell lines, murine xenografts, and human adenocarcinoma specimens. *P. anaerobius* activates phosphatidylinositol 3-kinase-protein kinase B (PI3K)-Akt signaling via integrin α2β1 in ApcMin/+ mice, further enhancing glycolytic flux and altering nucleotide metabolism (Rubinstein et al., 2013; Long et al., 2019).

Trimethylamine N-oxide (TMAO) is a gut microbiota-derived metabolite of dietary choline and carnitine, which exemplifies the context-dependent nature of microbial metabolites in carcinogenesis. In preclinical studies of CRC, TMAO has a predominantly pro-carcinogenic effect through multiple mechanisms including the activation of the Wnt/β-catenin signaling, promotion of cellular proliferation through PI3K/AKT signaling, and promotion of angiogenesis through increased vascular endothelial growth factor (VEGF-A) production (Yang et al., 2022; Yan et al., 2025; Yang et al., 2025). TMAO also contributes to inflammation, oxidative stress, and endoplasmic reticulum stress (Zhou et al., 2024b; Saha et al., 2025). However, the relationship between TMAO and cancer risk may be more nuanced; the immunostimulatory role of TMAO in pancreatic cancer is discussed further in section 3.2.

### 2.2 Anti-carcinogenic mechanisms

In contrast, protective metabolites, predominantly SCFAs, counteract carcinogenesis through immune modulation, selective apoptosis induction, and epigenetic regulation (**Figure 2**). The primary fermentation products, acetate, propionate, and butyrate, are typically generated in an approximate 3:1:1 ratio and are produced by distinct bacterial groups: acetate by most enteric bacteria, propionate primarily via the succinate pathway by Bacteroidetes, and butyrate by Firmicutes, including *F. prausnitzii* and *Roseburia* species (Louis and Flint, 2017; Kircher et al., 2022). SCFAs, particularly butyrate and propionate, function as natural histone deacetylase (HDAC) inhibitors, promoting histone hyperacetylation that downregulates pro-inflammatory cytokines, including IL-6 and IL-12 in colonic macrophages *in vitro* and in murine colitis models (Chang et al., 2014; Lee et al., 2017). Furthermore, through G-protein-coupled receptor (GPCR) signaling (109A and 43), SCFAs promote regulatory T-cell differentiation and IL-10 production while blocking NF-κB activation as shown in human dendritic cell cultures, murine models, and human CRC tissue (Thangaraju et al., 2009; Arpaia et al., 2013; Kaisar et al., 2017; Carretta et al., 2021; Schiweck et al., 2022). Additionally, butyrate enhances CD8+ T cell function and the efficacy of anti- programmed cell death protein 1 (PD-1) immunotherapy through direct TLR-5 interactions in syngeneic mouse models, with supporting evidence from human CRC stool samples (Kang et al., 2023) and epigenetically increases histone acetylation through CD8+ cells, enhancing antitumor cytokine production (Luu et al., 2021; Zhu X. et al., 2023). Butyrate can selectively induce apoptosis in cancer cells while sparing normal cells. Normal colonocytes efficiently utilize butyrate, whereas cancer cells relying on the Warburg effect cannot, leading to intracellular accumulation, HDACs inhibition, and apoptosis a mechanism characterized in human colon cancer cell lines and further corroborated by human colon biopsy specimens (Hu et al., 2011; Donohoe et al., 2012; Hajjar et al., 2021; Salvi and Cowles, 2021; Feitelson et al., 2023). Butyrate can also reverse p53-mutation-induced genetic abnormalities by regulating the expression of chromosome segregation 1, thereby causing G1-phase cell cycle arrest *in vitro* and in murine CRC models (Chang et al., 2022). Propionate functions through different mechanisms, mediating p21 expression through the mitochondrial phosphoenolpyruvate carboxykinase (PEPCK-M) enzyme and mechanistic target of rapamycin complex 2 (mTORC2)/3-phosphoinositide-dependent protein kinase-1 (PDK1)/Akt signaling pathways in epithelial cancer cell lines, while both SCFAs activate activator protein-1 (AP-1) signaling to control cell proliferation and apoptosis (Nepelska et al., 2012; Astakhova et al., 2016; Son and Cho, 2023). Additionally, urolithin A, produced by *Enterocloster* species from dietary ellagitannins, induces p53-dependent apoptosis by inhibiting mouse double minute 2 homolog (MDM2)-mediated p53 degradation and promoting mitochondrial apoptosis pathways in prostate cancer cell lines (Mohammed Saleem et al., 2020). As natural HDAC inhibitors, SCFAs reactivate tumor suppressor genes silenced by hypermethylation by increasing histone acetylation at promoter regions (Sarkar et al., 2011; Miro-Blanch and Yanes, 2019; Sun et al., 2019; Luu et al., 2021).

Moreover, the gut microbiota shapes DNA methylation patterns through the production of S-adenosyl-methionine and α-ketoglutarate, essential cofactors for methylation reactions (Mischke and Plosch, 2016; Rubas et al., 2025). Tryptophan catabolites, including indole-3-lactic acid, indole-3-aldehyde, and indole-3-propionic acid, further enhance CD8+ T cell immunity by modifying chromatin accessibility at immune gene loci and promoting interferon (IFN)-γ production; specifically, indole-3-aldehyde (I3A) activates aryl hydrocarbon receptor signaling in tumor-infiltrating CD8+ T cells, while indole-3-propionic acid increases H3K27 acetylation at the Tcf7 super-enhancer region, promoting CD8+ T cell stemness and improving immunotherapy responsiveness across multiple cancer types (Bender et al., 2023; Zhang et al., 2023; Jia et al., 2024; Zhang X. et al., 2025) (**Figure 2****).**

In summary, the balance between pro- and anti-carcinogenic bacterial metabolites mediates cancer risk. Pro-carcinogenic metabolites, including SBAs, bacterial genotoxins, and inflammatory products, promote tumorigenesis through DNA damage, chronic inflammation, and metabolic reprogramming. Conversely, protective metabolites, particularly SCFAs, counteract these processes by enhancing immune surveillance, inducing selective apoptosis of cancer cells, and reactivating tumor suppressor genes via epigenetic mechanisms. See **Table 1**.

**Table 1:** Representative anti-carcinogenic microbial metabolites and mechanisms.

Anti-carcinogenic metabolites			
Metabolite class	Specific metabolites	Mechanism of action	Effect
Short-chain fatty acids	Acetate	• HDAC inhibition • GPR109A/GPR43 signaling • Blocks NF-κB activation	Anti-inflammatory, promotes Treg differentiation
	Propionate	• HDAC inhibition • Mediates p21 expression via PEPCK-M enzyme • mTORC2/PDK1/AKT signaling • Activates AP-1 signaling • GPR109A/GPR43 signaling	Cell cycle arrest, controls proliferation/apoptosis, and IL-10 production
	Butyrate	• HDAC inhibition • GPR109A/GPR43 signaling • Enhances CD8+ T cells • Reactivates tumor suppressor genes	Selective cancer cell apoptosis, immune enhancement, G1 phase arrest, and enhances anti-PD-1 therapy
Tryptophan catabolites	Indole-3-lactic acid	• Modifies chromatin accessibility at immune gene loci • Promotes IL-12a production	Enhanced CD8+ T cell immunity
Polyphenol Metabolites	Urolithin A	• Inhibits MDM2-mediated p53 degradation • Promotes mitochondrial apoptosis pathways	p53-dependent apoptosis
Methylation Cofactors	S-adenosyl-methionine	• Essential cofactor for methylation reactions • Influences DNA methylation patterns	Epigenetic regulation of oncogenes and tumor suppressors

## 3 Examples of microbial metabolomics in specific malignancies

In light of the dual roles of microbial metabolites in carcinogenesis, this section summarizes the role of metabolomics across cancer-type specific evidence spanning colorectal, pancreatic, breast, liver, and head and neck malignancies.

### 3.1 Colorectal cancer

CRC is the third most common cause of cancer in men and women globally (Zhou and Rifkin, 2021).CRC is thought to originate from intestinal stem or stem-like cells that progressively lose tumor-suppressor function and acquire oncogenic properties through the accumulation of genetic and epigenetic alterations. Understanding the regulatory pathways that drive their growth represents a promising avenue for the development of preventive and therapeutic strategies (Testa et al., 2018). Various studies have shown the link between CRC, the gut microbiota, and the metabolites it produces. Notably, the impact of gut microbiome spans all age groups, influencing inflammation, innate immunity, and cognitive function. *In vivo* and *in silico* data showed strong distinctions in the gut microbial composition between young and elderly individuals. Remarkably, unique microbial metabolites, such as beneficial BAs, accumulate in centenarians, where they may act as ‘gut hormones,’ and serve as potential biomarkers of longevity and disease resilience (Kong et al., 2023). Microbial dysbiosis is a hallmark of CRC and contributes to chronic inflammation and variations in therapeutic response. Although gut microbes communicate with the host through metabolites, the specific roles of these metabolites in CRC are still poorly understood (Kang et al., 2025). Fecal metabolites were studied in mouse models of colon tumorigenesis with different mutational burdens. Data showed that fecal metabolites from healthy mice and humans suppress tumor cell growth; however, this effect is attenuated in CRC-bearing hosts (Bell et al., 2022). Microbiome analysis showed consistent reduction of *Lactobacillus reuteri* and its metabolite, reuterin, in both murine and human CRC. Reuterin disrupts redox homeostasis and reduces proliferation and survival in colon cancer cells by impairing protein oxidation, ribosomal biogenesis, and protein translation. *In vivo* supplementation of exogenous *L. reuteri* restricts colon tumor growth, increases tumor reactive oxygen species, and decreases protein translation. These findings indicate that a healthy microbiome, specifically *L. reuteri*, protects against CRC through microbial metabolite signaling (Bell et al., 2022). Moreover, SCFA microbial metabolites have been studied in relation to colorectal health and may play a protective role against CRC. Combined analysis of acetic, propionic, and butyric acids revealed that individuals at high risk for CRC have lower concentrations of these SCFAs (Alvandi et al., 2022). Similarly, reduced SCFA levels were observed in CRC patients compared with healthy controls. Qualitative analyses further showed that the majority of studies reported significantly lower fecal concentrations of SCFAs in individuals at higher risk of CRC while a substantial proportion of studies found lower fecal levels in patients compared to healthy individuals (Alvandi et al., 2022). These findings highlight the potential protective role of SCFAs in colorectal health.

Consistent with these observations, large-scale fecal metabolomic studies in CRC cohorts have documented depletion of butyrate-producing bacteria, including *Faecalibacterium prausnitzii* and *Roseburia intestinalis*, accompanied by reduced fecal butyrate concentrations (Dikeocha et al., 2022; Obuya et al., 2022). This depletion compromises colonocyte energy supply, weakens epithelial barrier integrity, and diminishes immunosurveillance, collectively creating a permissive microenvironment for CRC initiation and progression.

In parallel, stool metabolomic studies have reported elevated levels of SBAs, particularly DCA and LCA, in CRC patients compared with matched healthy controls. Experimental studies demonstrate that DCA can activate oncogenic signaling pathways, including Wnt/β-catenin and NF-κB, in CRC cell lines and patient-derived organoid models (Pai et al., 2004; Ma et al., 2022).

Additionally, hydrogen sulfide (H_2_S), produced by sulfidogenic bacteria such as *Desulfovibrio* species and *Bilophila wadsworthia*, represents another microbial metabolite implicated in CRC pathogenesis. A prospective cohort study involving 51,529 men in the Health Professionals Follow-up Study demonstrated that long-term adherence to a sulphur-metabolising dietary pattern was associated with a 43% increased risk of distal CRC and adjusted risk ratio of 1.43 (p-trend= 0.002) (Nguyen et al., 2020). Metagenomic analysis of 667 participants further confirmed enrichment of sulfidogenic genes from *Desulfovibrio* and *Bilophila* in CRC tissues (Wolf et al., 2022).

Collectively, these findings highlight how alterations in microbial metabolites including SCFAs, BAs, and H_2_S contribute to colorectal carcinogenesis and may provide promising biomarkers and therapeutic targets for CRC prevention and treatment.

### 3.2 Pancreatic cancer

Pancreatic cancer is one of the leading causes of cancer-related death worldwide (Klein, 2021). In pancreatic ductal adenocarcinoma (PDAC), disruption of microbiome homeostasis has been associated with alterations in the TME and advancement of oncogenesis through immune suppression, with implications for response to therapy and patient survival (Irajizad et al., 2023). A recent study developed a three-marker panel of microbial-derived metabolites to estimate 5-year pancreatic cancer risk. The panel comprised TMAO, indoleacrylic acid, and an additional indole derivative, each independently associated with future pancreatic cancer development (Irajizad et al., 2023). Incorporation of five additional non-microbial metabolites further enhanced predictive performance. When combined with carbohydrate antigen 19-9 (CA 19-9), a well-established blood-based biomarker for pancreatic cancer, individuals in the top 2.5th percentile of the composite metabolite plus CA 19–9 score had absolute 5-year risk estimates exceeding 13%. Together, these findings suggest that metabolite-based risk stratification may offer a promising approach for identifying high-risk individuals who could benefit from targeted surveillance or early interception strategies (Irajizad et al., 2023). A plethora of data suggests that gut flora is linked to immune regulation and improved response to immune checkpoint blockade (ICB). Although the underlying mechanisms remain unclear, microbial metabolites are thought to play a significant role (Mirji et al., 2022). A research study demonstrated that gut microbe-derived metabolite TMAO heightened anti-tumor immunity to PDAC via non-targeted LC-MS/mass spectrometry (MS) metabolomic screening. Administration of TMAO intraperitoneally or via dietary choline supplementation suppressed tumor growth in orthotopic PDAC mouse models, correlating with an immunostimulatory tumor-associated macrophage phenotype and activated effector T cell response in the TME. Functionally, TMAO potentiated the type-I IFN pathway and exerted anti-tumor effects in a type-I IFN-dependent manner (Mirji et al., 2022).

In addition to TMAO, has also been implicated in modulating therapeutic responses in PDAC. The microbiota-derived metabolite indole-3-acetic acid (3-IAA) has been identified as a modulator of chemotherapy response, with Tintelnot et al. showing that 3-IAA was enriched in fecal samples of PDAC patients responding to FOLFIRINOX therapy (Tintelnot et al., 2023). In gnotobiotic PDAC mouse models, fecal microbiota transplantation from responders or oral supplementation with 3-IAA enhanced the antitumor efficacy of FOLFIRINOX. Mechanistically, 3-IAA is oxidized by neutrophil myeloperoxidase, leading to downregulation of antioxidant enzymes (GPX3 and GPX7), increased intratumoral oxidative stress, and impaired protective autophagy in cancer cells, thereby sensitizing tumors to chemotherapy.

Probiotics are live microorganisms that confer health benefits and are increasingly explored for their potential role in cancer therapy through modulation of the gut microbiome. In pancreatic cancer models, *Lactobacillus casei* and *L. reuteri* altered gut microbial composition by reducing *Alloprevotella* and increasing fecal azelate and glutamate levels. Mechanistically, these probiotics inhibited TLR-4 expression in pancreatic cancer cells and promoted M1 macrophage polarization in the TME, suggesting that their combined use may represent a promising therapeutic strategy for pancreatic cancer (Zhu Z. et al., 2023). Ren et al. (2017) reported that pancreatic carcinoma patients exhibit reduced gut microbial diversity, including a decrease in butyrate-producing bacteria. Since butyrate-producing bacteria are major contributors to SCFA production, their reduction suggests a potential decrease in beneficial SCFAs, therefore suggesting lower levels of protective SCFAs (Ren et al., 2017). Zhou et al. (2021) confirmed these findings in PDAC patients, showing reduced butyrate-producing bacteria (*F. prausnitzii*, *Eubacterium rectale*, *R. intestinalis*), depletion of SCFA synthesis pathways, and decreased fecal butyrate levels (Zhou et al., 2021). Functionally, Panebianco et al. (2022) demonstrated that butyrate inhibits pancreatic cancer cell growth and enhances gemcitabine efficacy *in vitro* and *in vivo*, highlighting its potential anti-tumorigenic role beyond microbial production (Panebianco et al., 2022). Together, these studies suggest that reduced butyrate, one of the major SCFAs produced by gut bacteria, may link gut dysbiosis to pancreatic cancer pathogenesis, progression, and therapy response. Lastly, in a prospective study of advanced pancreatic cancer patients, plasma metabolomic profiling revealed that metabolic intermediates differed according to chemotherapy response (Muranaka et al., 2023). Notably, patients showing progressive disease after gemcitabine-based chemotherapy exhibited increased levels of BA metabolites. In the PANCAX−1 study, the specific conjugated BA elevated in these patients was taurocholic acid, these findings suggest an association between altered BA metabolism and pancreatic cancer progression or therapy response and highlight the potential of BA–related metabolic signatures as biomarkers for predicting chemotherapy outcome (Muranaka et al., 2023).

### 3.3 Breast cancer

Breast cancer (BC) is the most frequently diagnosed cancer worldwide (11.7% of all cases), with 70% of cases occurring in women without established risk factors (Nandi et al., 2023). In BC patients, the gut microbiota is significantly altered compared to healthy individuals, suggesting a potential link between specific microbes and breast cancer development and therapeutic responses. Studies comparing women with benign breast lesions, breast cancer, and control groups revealed heightened levels of *Porphyromonas* and *Peptoniphilus* in the BC group. At the same time, *Escherichia* and *Lactobacillus* species were enriched in women with benign breast lesions. Microbiota community richness was higher in benign groups than in malignant groups, with overall lower microbial diversity observed in cancer patients versus healthy controls (Nandi et al., 2023). BAs and their metabolites can reach breast tumors via BA transporters, where they may reduce tumor aggressiveness and improve prognosis, partly by influencing hormonal pathways. However, secondary BAs display context-dependent roles: For instance, ursodeoxycholic acid (UDCA) has been shown to reduce tumor aggressiveness and improve prognosis by modulating hormonal pathways, whereas certain SBAs, such as DCA and LCA, may promote carcinogenesis through DNA damage, β-catenin activation, and immunosuppressive effects (Guo et al., 2025). In BC, bacterial groups such as *Clostridium, Bacillus, Lactobacillus, Streptococcus*, and *Proteobacteria* are proficient in protein metabolism. In BC cells, tryptophan breakdown is suppressed, and this reduced catabolism is associated with poorer survival. Conversely, high extracellular tryptophan levels correlate with worse prognosis, suggesting that both intracellular metabolism and systemic availability are important for disease progression (Guo et al., 2025). Microbiota-derived tryptophan metabolites, such as indole-3 propionic acid (IPA) and indoxyl sulfate, can influence tumor behavior and immune function. These metabolites act, in part, via aryl hydrocarbon receptor (AHR) activation, modulating antitumor immunity and potentially exerting cytostatic effects on breast cancer cells. This highlights that, similar to BAs, individual tryptophan metabolites may have distinct and context-dependent effects on tumor growth and patient outcomes (Guo et al., 2025). A study demonstrated that in triple-negative BC, increased kynurenine pathway activity and depletion of purine metabolites impair CD8^+^ T-cell function, contributing to immunotherapy resistance (Sarfraz et al., 2024). Finally, this complex and context-dependent interplay between BC, gut microbiota, and microbial metabolites underscore the critical need for further studies to delineate specific mechanisms and identify therapeutic opportunities.

### 3.4 Liver cancer

Liver cancer is the fourth leading cause of cancer-related deaths worldwide and typically develops in the context of cirrhosis and chronic inflammation (Li et al., 2021). The liver preserves systemic homeostasis through functional zonation within hepatic lobules, whereby hepatocytes perform distinct metabolic tasks according to their spatial location. This tightly regulated spatial and temporal gene expression is essential for normal liver function but becomes disrupted during liver disease (Li et al., 2021). Importantly, hepatic microenvironments are influenced by gut microbiota–derived metabolites, which can modulate inflammation and metabolic zonation, thereby potentially contributing to the initiation and progression of liver cancer.

Advances in metabolomics, including metabolic profiling, expanded metabolome coverage, and efforts to improve reproducibility at every analytical stage—aim to identify common molecular signatures across liver conditions such as fatty liver disease, hepatitis, and cirrhosis. However, detecting these shared metabolites remains challenging due to the liver’s cellular diversity and the complexity of its metabolome (Ganesan et al., 2022).

The Singapore Chinese Health Study demonstrated that elevated pre-diagnostic circulating levels of SBAs were significantly associated with an increased risk of hepatocellular carcinoma (HCC) (Thomas et al., 2021). In particular, higher concentrations of DCA and other SBAs were linked to greater HCC risk, independent of established risk factors. These findings support the role of BA dysregulation in hepatocarcinogenesis and suggest that circulating BA profiles may serve as potential biomarkers for early HCC risk stratification (Thomas et al., 2021).

Complementary data from the REVEAL cohort showed that elevated glycocholic acid increased HCC risk in hepatitis B (HBV)- and hepatitis C (HCV)-related disease, while DCA was inversely associated with HBV-HCC (Petrick et al., 2020). Mechanistically, BAs regulate hepatic anti-tumor immunity through gut microbiota–dependent pathways: primary BAs promote CXCL16 expression and recruitment of CXCR6^+^ NKT cells with anti-tumor activity, whereas SBAs suppress this pathway and facilitate tumor growth (Ma et al., 2018). More recently, primary BAs were shown to induce oxidative stress in CD8^+^ T cells, and LCA inhibits T-cell activity, while modulation of BA metabolism can enhance responses to immune checkpoint therapy (Varanasi et al., 2025).

Gut microbiome dysbiosis contributes to the development of alcohol-related liver disease, and the microbial metabolite urolithin A (UA) has shown promise in alleviating liver injury, although its protective mechanisms remain incompletely defined (Zhang H. et al., 2024). One study demonstrated that UA mitigates alcohol-induced metabolic disturbances and hepatic endoplasmic reticulum stress via a gut–microbiota–liver axis involving major urinary protein 1 (MUP1). UA also restored alcohol-induced microbial imbalance by increasing the abundance of *Bacteroides sartorii*, *Parabacteroides distasonis*, and *A. muciniphila*, along with enhancing production of their metabolite propionic acid (Zhang H. et al., 2024). Although antibiotic-induced microbiota depletion partially diminished UA’s hepatoprotective effects, fecal microbiota transplantation (FMT) from UA-treated mice reproduced similar benefits through modulation of MUP1 (Zhang H. et al., 2024).

Another study investigating early recurrence of HBV-related HCC after surgery analyzed stool samples from 124 patients with HBV-associated HCC, 82 patients with HBV-related hepatitis, and 86 healthy controls using 16S rRNA sequencing, along with targeted metabolomics of 35 tumor tissues (Zheng et al., 2023). The findings revealed that specific gut microbes, including *Dialister*, *Veillonella*, and *Lactobacillus*, as well as 23 metabolites such as acetic acid, were associated with early postoperative recurrence. The authors proposed a gut–liver metabolic axis in which gut-derived acetic acid may promote tumor regrowth. A predictive model integrating microbial and clinical markers achieved an area under the curve (AUC) of 78.0%, underscoring the microbiome’s potential as a biomarker for recurrence risk (Zheng et al., 2023).

Obesity is a well-established risk factor for several cancers, including HCC. One study demonstrated that obesity-induced hepatic translocation of lipoteichoic acid (LTA), a Gram-positive bacterial component, promotes hepatocarcinogenesis (Loo et al., 2017). In combination with the obesity-associated microbial metabolite DCA, LTA enhances the senescence-associated secretory phenotype in hepatic stellate cells, leading to cyclooxygenase-2 upregulation via TLR-2 signaling (Loo et al., 2017). This process increases prostaglandin E2 (PGE2) production, which suppresses anti-tumor immunity. Similar mechanisms were observed in human non-alcoholic steatohepatitis-associated HCC, suggesting that targeting PGE2 signaling may represent a potential therapeutic strategy (Loo et al., 2017).

Overall, these studies underscore the critical role of gut microbiota–derived metabolites, particularly BAs, in modulating liver cancer risk, progression, and therapy response, highlighting their potential as biomarkers and therapeutic targets.

### 3.5 Head and neck cancers

Growing evidence underscores the influence of the microbiota–metabolite axis in head and neck cancers (HNC). In oral squamous cell carcinoma (OSCC), one study demonstrated that tumor-colonizing *Streptococcus mutans* reprograms the TME by inducing high levels of kynurenine via its surface adhesion protein antigen C. This activates the AHR, promotes neutrophil infiltration, drives CD8^+^ T-cell exhaustion, and leads to poor response to PD-1/programed death-ligand 1 (PD-L1) blockade (Zhou J. et al., 2024).

Periodontitis-associated microbiota has also been implicated in OSCC progression. Activation of γδ T-cells by these microbial communities accelerates tumor development. Notably, inhibition of γδ T-cells altered gut microbial and metabolic profiles, enriched *Lactobacillus murinus*, and linked these changes to adenine metabolism and IL-17 signaling. These findings suggest the existence of a gut–oral axis contributing to periodontitis-driven OSCC (Wei et al., 2024). Another group showed that psychological stress further shapes the microbiota–metabolite landscape in HNC. Chronic restraint stress was shown to reshape the oral microbiota, enriching *Pseudomonas* and *Veillonella* while depleting commensals such as *Corynebacterium* and *Staphylococcus*. This dysbiosis increased systemic kynurenine levels, stabilizing AHR in tumor-reactive CD8^+^ T cells, thereby promoting immune exhaustion and progression of head and neck squamous cell carcinoma (HNSCC) (Lou et al., 2025).

Therapeutic modulation of the microbiome has also been explored. The ROMA-2 trial investigated gut microbiome modulation in patients with human papillomavirus -related oropharyngeal squamous cell carcinoma undergoing chemoradiation (Oliva et al., 2024). Administration of the microbial ecosystem therapeutic 4 was feasible, safe, and well tolerated. Exploratory analyses suggest metabolomic shifts and ecological responses, particularly in stage III patients, supporting the potential of microbiome-based adjunct therapies (Oliva et al., 2024).

Distinct gut microbes and metabolites have additionally been associated with treatment response in nasopharyngeal carcinoma. Non-responders exhibited enrichment of *Bacteroides acidifaciens* and elevated acetate levels, whereas responders showed higher abundance of *Propionibacterium acnes* and *Clostridium magna* (Liu et al., 2025). Lastly, dietary interventions may further influence microbial metabolite signaling in HNC. One study demonstrated that time-restricted eating (TRE) modulates the gut microbial metabolome by enriching *Clostridium butyricum*, increasing fecal butyrate and tryptophan metabolites while reducing their systemic levels (Bari et al., 2024a). These metabolic changes correlated with prolonged progression-free survival. Mechanistically, reduced circulating butyrate and 5- hydroxyindoleacetic acid relieved AHR-mediated suppression of CD8^+^ T cells, thereby enhancing antitumor immune responses to ICB (Bari et al., 2024a). Collectively, these findings illustrate diverse yet convergent mechanisms through which microbial metabolites regulate immune function, influence therapeutic response, and drive tumor progression across multiple HNC subtypes. Moreover, these data suggest that HNCs represent only one component of a broader and still understudied spectrum of malignancies in which microbiota–metabolite interactions may play a critical role, warranting further dedicated investigation.

## 4 Clinical applications of microbial metabolomics in oncology

### 4.1 Microbial metabolites as diagnostic and prognostic biomarkers

Tumor cells shape their surrounding microenvironment through signaling that promotes growth and metastasis. Many cancers express specific markers; some markers can give insights into a patient’s cancer progression and potential responses to therapies. Microbial metabolites are emerging as additional diagnostic and prognostic biomarkers. Within tumors, these metabolites act as ligands or cytokines that modulate protein activation and influence the TME. For example, high levels of ammonia in the intestine can serve as a prognostic biomarker in colon cancer, as microbial metabolism increases T cell exhaustion, which leads to decreased proliferation. Studies have found an increase in lactate accumulation, leading to a reduced immune response to the infiltrating tumor cells (Zhou et al., 2024a). The buildup signals autocrine and paracrine mechanisms that activate GPCR81, which stimulates angiogenesis, a hallmark of cancer progression. Lipopolysaccharide (LPS) is part of the cell wall in Gram-negative bacteria that can cause inflammation and high expression of PD-1/PD-L1 (Li et al., 2024). Myeloid-derived suppressor cells and regulatory T cells can also accumulate, giving the TME the ability to suppress the body’s immune system. Thus, LPS can serve as a marker of cancer advancement (Li et al., 2024). SCFAs display both anticancer and cancer-promoting properties. *Lactobacillus* is associated with a positive response to anti-PD-1 therapy in gastrointestinal cancers due to their SCFA production. SCFAs have an important role in immune regulation, specifically being able to regulate regulatory T cells and IL-17 positive T cells (Xing et al., 2024). Recent CRC studies have explored different diagnostic and prognostic markers. *F. nucleatum* levels have been used to predict patient diagnosis, finding that the highest levels were found in stage IV patients (Sun et al., 2025). In gnotobiotic mice, *F. nucleatum* produces SCFAs that heighten inflammation and elevate CRC risk by increasing colonic Th-17 cells and inducing IL-17A and IL-17F expression (Li et al., 2024). This bacterium can also promote progression in BC as well as CRCs by traveling through the bloodstream or lymphatic system (Yao et al., 2026). Another study showed that esophageal squamous cell carcinoma patients with poor chemotherapy response had elevated *F. nucleatum* levels, suggesting a role in chemoresistance (Li et al., 2024). Elevated levels of *F. nucleatum* and *Porphyromonas gingivalis* could be used to assist early diagnosis of HNSCC. These two bacteria can promote cancer by invading epithelial cells and inhibiting apoptosis. However, the accumulation of *P. gingivalis* can be prevented and mitigated by using probiotics or microbiota modulating agents to target microbiota in the mouth (Yao et al., 2026). See **Supplementary Table 3**.

### 4.2 Modulation of cancer therapy efficacy and toxicity (chemotherapy and immunotherapy)

#### 4.2.1 Chemotherapy

Chemotherapy is widely regarded as a treatment for cancer, but has limitations, including variable patient response, potential resistance, and side effects that disrupt the microbiota. Irinotecan, used for multiple cancers, can cause toxicity by increasing β-glucuronidase (GUS) activity, which prolongs drug retention, damages the intestinal mucosa, and disrupts gut flora (Zhao et al., 2023). Thus, irinotecan-induced toxicity in some patients reduces the drug’s efficacy (Perez Escriva et al., 2025). Irinotecan has also been linked to inflammatory bowel disease. The intestinal microbe ecosystem becomes disrupted when irinotecan activates the immune system so intestinal vacuoles excrete increased levels of mucin. This reduces nutrient availability to the microbiota, leading to disruption. Severe diarrhea is also included in the side effect profile of irinotecan when GUS activity is enhanced. However, GUS activity was inhibited in xenograft mouse models and showed that this blocked enterotoxicity without modifying irinotecan’s efficacy (Sun et al., 2025). Adverse effects from docetaxel-based chemotherapies were mitigated by adding probiotics into patient diets. Additional probiotics were found to preserve the diversity of the gut microbiome (Zhou et al., 2025).

*Lactobacillus* can mitigate chemotherapy-induced mucositis and diarrhea, such as in oxaliplatin-treated CRC, by downregulating NF-κB, tumor necrosis factor (TNF)-α, and IL-6, helping counteract inflammation commonly exacerbated by cancer therapies (Li et al., 2024). Solid tumors are widely treated with cyclophosphamide (CTX), but it can cause acute mucosal injury. When combined with *Lactobacillus*, T cells are turned into Th-17 cells, which improves the effects of chemotherapy in melanoma and sarcoma mice. By itself, *Lactobacillus plantarum NCU116* improved intestinal mucosal injury from CTX by helping to regulate intestinal functions. This includes increasing SCFA levels and the population of lactic acid bacteria in the mouse intestine. The combination of cisplatin and *Lactobacillus* improves the response to therapy by activating pro-apoptotic genes to increase the serum levels of IL-6 and IFN-γ in tumor tissues (Sun et al., 2025). Intestinal epithelial cells have a peroxide decomposing enzyme, glutathione peroxidase (GPX-1 and GPX-2), which protects cells from peroxide damage by promoting the decomposition of hydrogen peroxide. *Helicobacter pylori* inhibits the glutathione peroxidase function, thus suggesting that their presence can improve chemotherapy efficacy. Down-regulation of GPX-1 and GPX-2 in cancer mice was also shown to increase their sensitivity to chemotherapy (Sun et al., 2025).

Microbiota composition varies among cancer patients, particularly after treatment, suggesting that it can influence chemotherapy efficacy (Zhao et al., 2023). Gut-derived butyrate enhances oxaliplatin efficacy by regulating CD8^+^ T cells, whereas germ-free or antibiotic-treated mice exhibit reduced response, highlighting the potential of microbiota-targeted interventions (Zhao et al., 2023). A study of colon cancer patients who responded to chemotherapy had increased butyrate levels suggesting a potential chemotherapy-sensitizing effect (Li et al., 2024). Butyrate accumulation inhibits HDACs, leading to histone hyperacetylation, which reduces proliferation and promotes apoptosis in colonic and breast cancer cells (Perez Escriva et al., 2025). Moreover, polyphenolic metabolites can repair DNA and inhibit inflammatory signaling to promote anti-tumor effects, and UA sensitizes cancer cells to chemotherapy by downregulating drug transporters (Li et al., 2024).

#### 4.2.2 Immunotherapy

Immunotherapy remains a key cancer treatment, using immune checkpoint inhibitors (ICIs) targeting PD-1/PD-L1 to stimulate T-cells, and prevents tumors from escaping the immune landscape. Patients have varying immunotherapy outcomes; some respond very well, while others have little to no response. The gut microbiota influences host immunity, suggesting microbes may modulate immunotherapy efficacy (Abid and Koh, 2019; Zhao et al., 2023). Gut health is critical, as microbes aid digestion and absorption. Vitamin B5, found in intestinal bacteria and foods, enhances antitumor signaling via IL-22 producing T-cells and improves immunotherapy responses (Zhao et al., 2023). Mice treated with pantothenate had an improved efficacy of anti-PD-L1 therapy. High pretreatment plasma pantothenic acid levels in melanoma patients were also found to have an optimistic anti-PDL1 response (Xing et al., 2024).

A study showed that introducing *Lactobacillus johnsonii* bacteria into the gut supports immunotherapy responses by increasing CD8+ T cells. However, the response depended on the tryptophan metabolic pathway (Jia et al., 2024). Furthermore, the administration of IPA, a tryptophan-related metabolite in mice, increased CD8+ T cells and cytokine secretion, enhancing immunotherapy (Jia et al., 2024). In BC patients, IPA levels are suppressed. IPA has been shown to reduce cancer stem cell populations, inhibit cellular proliferation, and inhibit epithelial-mesenchymal transition, thereby enhancing antitumor response (Zhou et al., 2025). Similarly, a mouse melanoma study showed that *L. reuteri* releases I3A in the TME, activating AHR signaling in CD8^+^ cells and boosting anti-PD-L1 efficacy. Germ-free mice treated with cytotoxic T-lymphocyte associated protein-4 (CTLA-4)-blockade had less response compared to those with a gut microbiome. The germ-free mice had their therapy response restored when certain bacteria were supplemented. *Bifidobacterium* strains enhanced immune functions and increased tumor infiltration, thus increasing anti-PD-1 efficacy (Xing et al., 2024). *Bifidobacterium pseudolongum*-derived acetate could inhibit the IL-6/JAK/STAT3 pathway, thereby limiting tumor progression. In ovarian and breast cancer, increased acetate via *A. muciniphila* or supplementation and enhances CD8^+^ T cell antitumor activity (Song et al., 2025).

In mice, antitumor immunity and anti-PD-1 efficacy were improved by the production of TMAO. As mentioned before, TMAO does this by increasing the activation of IFN-γ, TNF-α, and CD8 + T cells. Higher levels of TMAO and CD8+ T cells were found in mice that were fed a choline-rich diet. However, the administration of a trimethylamine-lyase inhibitor (cutC inhibitor) reversed the positive effects of TMAO (Zhou et al., 2025). TMAO promotes macrophages and CD8+ T cells to infiltrate the TME, increasing the efficacy of ICIs. TMAO improved ICB efficacy in pancreatic mouse models (Xing et al., 2024). Lastly, D-lactate dimers (DLAD) have been shown to induce cytotoxicity in cancers. In melanoma, DLAD treatment caused complete cell damage, reduced xenograft size and proliferation, and enhanced innate immune responses, leading to decreased tumor growth (Gu et al., 2022).

Butyrate supplements increased the efficacy of oxaliplatin-based chemotherapies and anti-PD-L1 immunotherapy. In a mouse study, mice treated with butyrate from R. intestinalis showed enhanced antitumor activity and enhanced immunotherapy response, though it may also have some tumor-promoting effects. In germ-free mice, treatment with *Lachnospiraceae* impaired radiotherapy response despite increased butyrate levels. Some microbial-derived butyrate can inhibit IFN genes and the anti-CTLA-4 pathway, limiting tumor-specific CD8^+^ T cell responses and reducing immunotherapy efficacy (Song et al., 2025).

## 5 Therapeutic targeting of the microbiome-metabolite axis

### 5.1 Diet

There have been many research avenues exploring dietary interventions to modulate the gut microbiome, but fewer efforts to modulate the metabolites. One novel approach has been utilizing time restricted eating (TRE) to improve response to ICB therapy. In a phase I/II nonrandomized study, patients with metastatic HNSCC receiving a single agent ICB were assigned to a 14 hour fast (TRE) vs. no dietary intervention. Following a 12 +/- 3 week period, patients who were assigned to TRE had significantly improved disease control rate at 3 and 6 months (p<0.001 and p=0.02, respectively) as well as lower immunosuppressive indole metabolites such as butyric acid and tryptophan metabolites (Liu et al., 2025). In clear cell RCC, TRE similarly improved ICB response in mice, leading to longer progression-free survival than controls (Bari et al., 2024b). Future research should focus on isolating and characterizing UA–producing bacterial strains and developing them as candidate probiotic formulations (Yang et al., 2023). Evaluating their efficacy in preclinical models and controlled clinical studies will be important to determine whether targeted microbial supplementation can enhance endogenous UA production and improve relevant metabolic or anticancer outcomes.

### 5.2 Prebiotics, probiotics, and postbiotics

Therapeutic modulation of the microbiome can be approached through three distinct but mechanistically linked strategies: prebiotics, probiotics, and postbiotics. These categories that are frequently conflated but operate at different points along the same biological axis (Al-Habsi et al., 2024). Understanding their relationship is essential for evaluating their respective therapeutic potential.

Prebiotics are non-digestible dietary substrates, primarily oligosaccharides and polysaccharides such as inulin, fructooligosaccharides (FOS), and galactooligosaccharides (GOS), that selectively promote the growth and activity of beneficial gut microorganisms without themselves entering systemic circulation. Their therapeutic effect is therefore indirect: by nourishing specific microbial populations, prebiotics alter the composition and metabolic output of the microbiome, which in turn modulates host immune and metabolic responses. Li et al. demonstrated this principle in syngeneic mouse models of both CRC and NRAS-mutant melanoma, showing that dietary inulin supplementation inhibited tumor growth and enhanced antitumor immune responses by enriching butyrate-producing taxa within *Clostridium* cluster XIVa. These effects that were abolished in germ-free mice, confirming the indispensable role of gut bacterial metabolism as the intermediary (Li et al., 2020). In melanoma, this prebiotic–immune axis has direct clinical relevance: Spencer et al. reported in a prospective observational cohort that melanoma patients with high dietary fiber intake had significantly better responses to anti-PD-1 checkpoint immunotherapy than those on low-fiber diets, with stool metabolomic profiling revealing elevated SCFA propionate as a candidate mediator of enhanced CD4+ and CD8+ T cell activation within the TME (Spencer et al., 2021).

Probiotics are live microorganisms that, when administered in adequate quantities, directly modulate the composition or metabolic activity of the gut microbiome. Their therapeutic effects are mediated through the metabolites they produce and the immune interactions they provoke. Supplementation with *Alistipes onderdonkii-*containing probiotics has shown benefit in CRC treatment; metabolomic analysis of samples from PD-1 and *A. onderdonkii*-treated mice demonstrated upregulation of 2-hydroxybutyric acid, metyrosine, alanine betaine, 2-methylbutyrylglycine, piperidine, and monoethyl malonic acid compared to the non-probiotic group, correlating with improvement in regulatory immune functions (Xin et al., 2025). In melanoma, Enterococcus species improved the effects of anti-PD-L1 therapy in mouse models through the generation of immune-active muropeptides and specialized peptidoglycan remodeling activity that stimulate host immune responses (Griffin et al., 2021). Importantly, probiotic effects are not uniformly beneficial: Spencer et al. observed that undirected use of commercially available probiotic supplements was associated with reduced ICI response in melanoma patients, underlining that the composition and context of probiotic administration critically determine outcome (Spencer et al., 2021).

Postbiotics are the downstream effectors of this axis, namely the bioactive metabolites and structural components produced when probiotics ferment prebiotic substrates. These include SCFAs (principally butyrate, propionate, and acetate), SBAs, indole derivatives, and purine nucleosides, all of which have been discussed in detail as mechanistic mediators of cancer risk and immune modulation in Sections 2 and 3. CRC patients demonstrate consistently lower SCFA concentrations than non-CRC patients (Yusuf et al., 2018), illustrating that postbiotic deficiency is a feature of the disease state rather than merely a correlate. The postbiotic inosine, produced by *B. pseudolongum*, directly demonstrates multi-cancer relevance: Mager et al. showed that inosine enhanced ICI efficacy across CRC, melanoma, and bladder carcinoma mouse models through adenosine A2A receptor activation in T lymphocytes — an effect dependent on the microbiome’s metabolic activity and absent when the microbial source was removed (Mager et al., 2020).

Taken together, these three strategies are most productively understood not as alternatives but as a hierarchy: prebiotics provide the substrate, probiotics provide the enzymatic machinery, and postbiotics are the functional output that directly interfaces with host tumor biology and immune responses. Therapeutic strategies that target only one level of this axis, either supplementing a probiotic without attending to its prebiotic substrate or measuring postbiotic levels without attributing them to the responsible microbial community, will inevitably produce inconsistent results. Future clinical development in this space should increasingly adopt symbiotic approaches that co-administer defined prebiotic substrates with rationally selected probiotic strains, with postbiotic output as the measurable therapeutic endpoint.

### 5.3 Fecal microbiota transplantation

FMT has been studied as a therapeutic approach for multiple gastrointestinal conditions, including ulcerative colitis and Clostridium difficile infection. The basis of FMT is to replace the microbiome with that of a healthy donor, thereby promoting the growth of beneficial flora and reducing opportunistic bacterial overgrowth (Yadegar et al., 2024). Transplantation is achieved via colonoscopy or enema, or from above through a nasoduodenal tube or oral capsules. With further investigations, a role for FMT in conjunction with immunotherapy has been shown to improve outcomes in several malignancies (Davar et al., 2021).

A phase I clinical trial in metastatic melanoma patients who had failed immunotherapy evaluated the effects of FMT from two donors. After colonscopic infusion and oral stool-microbiota capsules, patients received nivolumab (anti-PD-1 antibody) therapy. Favorable changes in the tumor microbiome were observed, leading to activation of anticancer immunity. Specific bacterial phyla, such as *Actinobacteria* and *Firmicutes*, were associated with an improved response to therapy. These findings support further metabolomic sequencing studies to clarify which metabolites enhance the effectiveness of PD-1 therapy (Davar et al., 2021).

In the phase II FMT-Luminate trial, FMT administered prior to ICI therapy improved treatment responses in patients with non-small cell lung cancer and melanoma. Microbiome analyses suggested that the benefit was associated with restructuring of the gut microbiome and depletion of bacterial taxa linked to immunotherapy resistance. These findings highlight FMT as a potential strategy to enhance the efficacy of cancer immunotherapy through modulation of the gut microbiome (Duttagupta et al., 2026).

One consideration against FMT involves safety profiles and adverse effects. In metastatic RCC, a study assessing combined FMT and anti–PD-1 therapy reported immune-related adverse events in 80% of patients, leading many to discontinue treatment. Although there was an overall risk reduction of 44% (95% CI 30–60), larger studies are needed to fully evaluate the risk–benefit profile of this therapeutic approach (Fernandes et al., 2022).

## 6 Methodological considerations and future challenges

For microbial metabolomics to transition from a promising research field to a pillar of clinical oncology, several significant technical and conceptual hurdles must be addressed.

### 6.1 Key analytical techniques: a comparative overview

The two workhorse technologies for metabolomics are MS and NMR spectroscopy.

#### 6.1.1 Mass spectrometry

MS-based metabolomics ionizes molecules and separates them by mass-to-charge ratio, typically coupled with chromatographic separation via Gas Chromatography (GC-MS) for volatile or derivatized metabolites, or LC-MS for a wider range of polar and non-polar compounds. The platform’s capacity to resolve microbial from host metabolic contributions was demonstrated by Zierer et al., who applied GC-MS to twin cohort fecal metabolomics and demonstrated that a significant fraction of metabolome variation is attributable to microbial composition rather than host genetics providing causality benchmarks that have since anchored the field (Zierer et al., 2018). Its clinical utility has been demonstrated with equal directness: Yi et al. performed untargeted serum metabolomics using Liquid Chromatography-High Resolution Mass Spectrometry in 62 pre-treatment CRC patients and 61 matched healthy controls and identified nine candidate biomarkers via Orthogonal Projections to Latent Structures Discriminant Analysis (OPLS-DA) modelling. Surgical resection was associated with partial metabolic reversion toward a healthy metabolic profile, which demonstrated in a single primary study that LC-MS can serve both as a detection tool and as a monitor of treatment response (Yi et al., 2023).

- Strengths: MS offers exceptional sensitivity, detecting metabolites at very low concentrations (femtomolar to attomolar). Its high resolution and throughput make it ideal for untargeted, discovery-based studies that profile thousands of metabolic features and generate new biomarker hypotheses (Johnson and Gonzalez, 2012; Aretz and Meierhofer, 2016; Marshall and Powers, 2017).
- Weaknesses: Quantification and identification remain major challenges. Absolute quantification is difficult due to matrix effects and ion suppression, where the presence of other molecules in a complex sample can alter the ionization efficiency of the target analyte. Thus, MS data is often reported as relative abundance. Furthermore, identifying a metabolic feature based solely on its mass-to-charge ratio value alone is a significant bottleneck, requiring extensive matching against spectral libraries that remain incomplete (Aretz and Meierhofer, 2016).

#### 6.1.2 Nuclear magnetic resonance spectroscopy

NMR spectroscopy measures the resonance of atomic nuclei in a strong magnetic field, providing detailed information about a molecule’s chemical structure and its atomic connectivity. The applied strength of NMR in cancer metabolomics has been demonstrated across multiple sample types. Lin et al. applied Proton NMR fecal metabolomics to 68 CRC patients stratified by clinical stage and 32 healthy controls, showing that every stage of CRC including early stage I/II could be clearly discriminated from healthy controls using OPLS-DA, with discriminating metabolites including reduced butyrate and acetate reflecting disrupted microbial fermentation (Marshall and Powers, 2017). Gu et al. (2019) extended NMR–based metabolomics to serum samples from 40 CRC patients, 32 patients with colorectal polyps, and 38 healthy controls. NMR metabolic profiling successfully stratified individuals across the adenoma–carcinoma continuum. Specifically, glycolytic pathway activation was more prominent in CRC, whereas pyruvate pathway activation was characteristic of polyp patients. These findings establish serum NMR profiling as a potential tool not only for CRC detection but also for discrimination of pre-cancerous lesions (Lin et al., 2016).

- Strengths: NMR is inherently quantitative, highly reproducible, and non-destructive, allowing the sample to be preserved for further analysis (Emwas, 2015). Sample preparation is minimal, making it the gold standard for accurate quantification of high-abundance metabolites and for *de novo* structural elucidation of unknown compounds (Nagana Gowda and Raftery, 2015).
- Weaknesses: The main limitation of NMR is its relatively low sensitivity, with detection typically in the low micromolar range, orders of magnitude less sensitive than MS. Lower spectral resolution can also lead to significant peak overlap in complex biological mixtures, complicating compound identification (Marshall and Powers, 2017).

Together, the two techniques are highly complementary. See **Table 2**. for a side-by-side comparison. MS is superior for discovering low-abundance metabolites, while NMR excels at accurately quantifying the high-abundance ones and confirming structures. An integrated approach, MS for discovery and NMR or targeted MS for validation, offers the most comprehensive view of the metabolome (Fernandes et al., 2022).

**Table 2:** Comparison of key analytical platforms in metabolomics.

Parameter	Mass spectrometry (MS)	NMR spectroscopy
Sensitivity	High (femtomolar to attomolar range)	Low (~1 μM limit)
Reproducibility	Moderate	Very High
Quantitative capability	Challenging (relative quantification is standard)	Excellent (absolute quantification)
Metabolite identification	Relies heavily on spectral libraries (bottleneck)	Excellent for structural elucidation
Sample preparation	More complex; requires chromatography/derivatization	Minimal

#### 6.1.3 Spatial metabolomics

Conventional MS and NMR metabolomics analyze bulk tissue homogenates or biofluids, irrevocably losing the spatial context of metabolite distribution within the tumor architecture. Spatial metabolomics, primarily implemented through matrix-assisted laser desorption/ionization mass spectrometry imaging (MALDI-MSI) and desorption electrospray ionization MSI, addresses this limitation by mapping hundreds of metabolites directly within intact tissue sections at near single-cell resolution. This enables simultaneous interrogation of the tumor core, invasive margin, and stromal compartments without requiring prior knowledge of target analytes. In CRC research, this capacity has yielded critical mechanistic insights: MALDI-MSI applied directly to CRC models has visualized intratumoral glucose gradients consistent with the Warburg effect, physically mapping glycolysis and TCA cycle metabolites to differentiate the metabolic programming of the tumor core from the surrounding non-cancerous margins (Zhang Y. et al., 2025).

The relevance to microbial metabolomics is direct: metabolites such as SCFAs, SBAs, and indole derivatives produced by intratumoral and luminal microbiota are not uniformly distributed within tumor tissue, and their biological effects are inherently compartment-dependent. Demonstrating this principle in a primary clinical context, recent spatial mass spectrometry imaging (air-flow-assisted desorption electrospray ionization-mass spectrometry) mapped the intact TME of CRCs and revealed that spatially resolved microbiota-derived immunostimulatory metabolites, including inosine, are topographically co-localized with specific lipid distributions. This directly linked localized metabolic signatures to the modulation of anti-tumor immune responses and disease progression (Weng et al., 2025). Integrating spatial metabolomics with metagenomics to attribute tissue-level metabolite signals to specific microbial taxa represents one of the most consequential methodological frontiers in the field, though technical challenges including limited spatial resolution for small polar metabolites, matrix interference at low mass ranges, and the absence of standardized tissue preparation protocols currently constrain routine application.

### 6.2 Major hurdles: from correlation to clinic

The path to clinical translation faces multiple challenges: establishing causality, managing inter-individual variability, and achieving standardization.

#### 6.2.1 Establishing causality

Most human microbial metabolomics studies are cross-sectional, reporting statistical associations—such as the elevation of a specific metabolite in patients with malignancies (Qian and Ho, 2020). While useful for hypothesis generation, these correlations do not establish causation. Advancing to validated mechanisms requires a multi-tiered framework integrating experimental models, such as gnotobiotic animals and human-derived organoid co-cultures with robust epidemiological designs. A major challenge is overcoming reverse causality inherent in cross-sectional data, necessitating population-level approaches that distinguish true causal pathways from confounding variables (Hu et al., 2024; Wilechansky et al., 2025).

Two complementary designs strengthen causal inference: prospective longitudinal cohort studies and Mendelian randomization (MR). Prospective studies with pre-diagnostic biospecimens address reverse causation by showing that metabolite alterations precede disease onset. In the UK Biobank, Buergel et al. applied deep neural networks to 168 NMR markers from 117,981 participants, predicting 24 disease outcomes, including cancers, with validation across four cohorts (Buergel et al., 2022). In the EPIC cohort, a pan-cancer analysis of 5,828 case-control pairs identified six metabolites inversely associated with cancer risk, suggesting shared metabolic vulnerability pathways (Breeur et al., 2022). In CRC, Vidman et al. analyzed 902 pre-diagnostic plasma samples collected up to 26 years before diagnosis, identifying seven metabolites linked to future risk and minimizing reverse causation (Vidman et al., 2023).

MR provides an orthogonal approach by using germline genetic variants as instrumental variables for metabolite levels, effectively mimicking randomized controlled trials. Liu et al. identified 58 causal microbiome–metabolite relationships, with 43 replicated across datasets (Liu et al., 2022). Using MiBioGen GWAS data from 18,340 individuals across 24 cohorts, Long et al. identified 11 causal links between microbiome genetic liability and eight cancer types (Kurilshikov et al., 2021; Long et al., 2023). For CRC, Yuan et al. combined 249 NMR metabolic biomarkers from 199,732 UK Biobank participants with MR analysis of 78,473 cases, representing the most statistically powered metabolomics MR study to date (Yuan et al., 2025). The strongest framework integrates prospective cohorts, MR, and mechanistic validation in organoid or gnotobiotic models, a standard the field should aim to achieve.

#### 6.2.2 Inter-individual variability

The human microbiome and metabolome are highly diverse, influenced by genetics, diet, medications, lifestyle, and other environmental exposures. This high degree of inter-individual “background noise” can obscure disease-related signals, making it difficult to identify consistent and universally applicable biomarkers from small-scale studies (Qian and Ho, 2020). Causal inference is strengthened by integrating metabolomic, metagenomic, and host genomic data within a structured multi-omics framework. Mechanistically, a metabolite associated with cancer risk in MR is only informative if its microbial biosynthetic pathway can be identified and its downstream host effects captured through transcriptomic or proteomic data. Without metagenomics, metabolites cannot be linked to specific taxa or gene clusters; without transcriptomics or proteomics, their effects remain correlative rather than mechanistic. This limitation is supported by empirical evidence. Gao et al. integrated fecal metagenomics with serum metabolomics in a 225-subject CRC cohort, showing that a combined microbe–metabolite panel outperformed either layer alone (Gao et al., 2022). Similarly, Kong et al. demonstrated that early- and late-onset CRC phenotypes were only distinguishable through integrated metagenomic and metabolomic analysis, remaining undetectable by either platform independently (Kong et al., 2023). Practically, integrated multi-omics analyses require harmonized sample processing, matched biobanking across omics platforms from the same individuals, and computational pipelines capable of handling cross-platform batch effects.

#### 6.2.3 Standardization

Protocols for sample collection, handling, storage, metabolite extraction, data acquisition, and analysis vary widely across laboratories, limiting comparability, meta-analysis, and statistical power. When rigorously applied, standardization yields substantial improvements: a targeted LC-MS study showed that standardized pretreatment, derivatization, and internal standards reduced intra- and inter-laboratory variance to below 10% for butyrate and other SCFAs (Liebisch et al., 2019). However, such consistency is not universal. Siskos et al. evaluated the AbsoluteIDQ p180 LC-MS platform across six laboratories using identical protocols but different instruments, finding that 18% of metabolites failed interlaboratory precision thresholds (CV <20%), with systematic variation in acylcarnitines and sphingomyelins (Siskos et al., 2017). The clinical impact is evident in cancer metabolomics. Cai et al. reported that no biofluid biomarker set has been consistently replicated across CRC studies, a limitation attributable to pre-analytical variability, instrument differences, and non-standardized data processing rather than biological inconsistency (Siskos et al., 2017; Cai et al., 2025). Addressing this requires three interdependent components: (1) pre-analytical standardization, defined collection tubes, processing within 30 minutes, −80 °C storage, and minimal freeze–thaw cycles (ISO 23118:2021); (2) analytical standardization—pooled quality controls, isotopically labeled internal standards, and NIST SRM 1950 reference plasma; and (3) data reporting, Metabolomics Standards Initiative (MSI) Level ≥2 annotation, raw data deposition in MetaboLights or Metabolomics Workbench, and transparent batch correction reporting. Although initiatives such as the MSI and the Metabolomics Quality Assurance and Quality Control Consortium have established these guidelines, adoption remains inconsistent in microbiome–cancer studies (Fiehn et al., 2007; Qian and Ho, 2020; Kirwan et al., 2022). Without these standards, cross-study meta-analysis of microbial metabolite–cancer associations, arguably the key pathway to clinical translation—remains unfeasible.

These challenges are interconnected, creating a self-reinforcing cycle. Progress requires shifting from small, isolated studies to large, multi-center investigations using harmonized protocols. Furthermore, greater emphasis on longitudinal studies, which track individuals over time, is needed to distinguish disease-relevant intra-individual changes from the background of stable inter-individual differences, providing a more powerful approach to establishing causality.

## 7 Conclusion and future directions

The microbial metabolome is no longer a peripheral consideration in cancer biology. It is a mechanistically active, clinically measurable, and therapeutically targetable dimension of oncogenesis. This review has traced that argument from foundational biology through analytical methodology to clinical application, documenting both the transformative potential and the unresolved challenges at each stage.

### 7.1 Where the field stands

Microbial metabolites operate as context-dependent effectors across the full spectrum of carcinogenesis; modulating DNA integrity, local and systemic inflammation, immune surveillance, and hormonal signaling. They do so not only in the gut but across distant organs through defined gut–organ axes. Metabolomic profiling is consolidating its role as a non-invasive clinical tool for diagnosis, prognostication, and therapy response prediction, while therapeutic strategies now span dietary intervention, FMT, postbiotics, and engineered living therapeutics. Despite this progress, three problems remain unresolved and rate-limiting: the gap between associative findings and mechanistic proof, the absence of standardized methodology that prevents reproducibility and cross-study synthesis, and insufficient integration of the analytical layers needed to move from metabolite signal to biological mechanism.

### 7.2 What must come next

The immediate priority is converting associative findings into mechanistic evidence, specifically for metabolite–cancer relationships flagged by MR but lacking resolution at the host cell level. The experimental infrastructure to do this exists, as discussed in Section 1.1.2; patient-derived organoids and gnotobiotic models have already demonstrated their capacity to isolate the causal contribution of individual metabolites to oncogenic programs. What is now required is their systematic deployment across the candidate pathways identified in Sections 2–5 that currently rest on epidemiological or correlative evidence alone.

The standardization and multi-omics integration frameworks required to make this field reproducible and scalable are addressed in Sections 6.2.2 and 6.2.3, and the spatial metabolomics platform that adds intratumoral anatomical resolution to those layers is characterized in Section 6.1.3. These methodological advances are not independent aspirations but interlocking prerequisites. Standardization enables cross-study data pooling; multi-omics integration attributes metabolite signals to specific taxa and host pathways; and spatial resolution localizes those signals to the tumor compartments where they are biologically consequential. No single layer is sufficient alone, and the field will not achieve clinical translation until all three operate in concert.

That convergence ultimately points toward a single clinical goal: routine pre-treatment microbial metabolomic profiling used to stratify patients for immunotherapy, guide personalized microbiome-priming interventions, and inform adjuvant therapy selection. Realizing this will require parallel evolution in clinical trial design, biobanking infrastructure, and regulatory frameworks capable of accommodating microbiome-based combination strategies. The patient in this paradigm is not merely a human genome but a holobiont, and oncology will only fully exploit the microbial metabolome when its clinical architecture is built to reflect that reality.
