# Supplementary material for: The intricate interplay of microbial metabolomics and carcinogenesis: a spotlight on mechanistic pathways, clinical implications and methodological challenges
Source: Front Cell Infect Microbiol. 2026 May 29;16:1787954. doi: 10.3389/fcimb.2026.1787954 (PMC13259796; doi:10.3389/fcimb.2026.1787954)
Supplement: Supplementary file 1 [file DataSheet1.pdf]

Supplementary Material

1 Supplementary Data

Supplementary Material should be uploaded separately on submission. Please include any supplementary data, figures and/or tables.

Supplementary material is not typeset so please ensure that all information is clearly presented, the appropriate caption is included in the file and not in the manuscript, and that the style conforms to the rest of the article.

2 Supplementary Figures and Tables

For more information on Supplementary Material and for details on the different file types accepted, please see here.

2.1 Supplementary Figures

Supplementary Table 1. Representative Pro-Carcinogenic Microbial Metabolites and Mechanisms

Pro-Carcinogenic Metabolites			
**Metabolite Class**	**Specific Metabolites**	**Mechanism of Action**	**Effect**
**Secondary Bile Acids**	**Deoxycholic acid**	• Generate ROS/RNS<br>• Increase DNA strand breaks<br>• Mitochondrial oxidative stress<br>• Activate MAPK via EGFR<br>• Disrupt mitochondrial membrane integrity	Promotes proliferation, invasiveness, enforces glycolysis, and creates an immunosuppressive microenvironment

	**Lithocholic acid**	• Increase MMP expression<br>• Increase urokinase-type plasminogen activator	Enhances cancer cell invasiveness
**Bacterial Toxins/Genotoxins**	**Colibactin**	• Alkylates DNA forming interstrand crosslinks<br>• Activates ATM/ATR-Chk2 pathway<br>• Activates Wnt/β-catenin signaling<br>• Accumulates p53 protein	Direct genotoxicity, tumor progression, especially in p53-mutant settings
	**Tilimycin**	• N-2 guanine alkylation	DNA damage
	**Cytolethal distending toxin**	• Acts as DNase<br>• Induces double-strand DNA breaks	Cell cycle arrest, apoptosis, and genomic instability
**Protein Fermentation Products**	**Trans-3-Indoleacrylic acid**	• Inhibits ferroptosis<br>• Upregulates ALDH1A3 expression	Creates ferroptosis-resistant phenotypes
	**Phenylacetic acid**	• Pro-inflammatory signaling	Chronic inflammation, pro-inflammatory cytokine expression
	**Phenols**		
	**Indoles**		
	**p-Cresol**		

**Hydrogen Sulfide (High Concentration)**	**H_2S**	• Genotoxic<br>• Impairs mitochondrial respiration<br>• Disrupts epithelial barrier<br>• Promotes AKT/ERK phosphorylation	Genotoxicity, impaired mitochondrial respiration, epithelial barrier disruption, and inflammation
**Other Pro-carcinogenic**	**Formate**	• Promotes the Warburg effect<br>• Integrates into one-carbon metabolism pathways	Enhanced nucleotide synthesis and proliferative capacity
	**TMAO**	• Activates Wnt/β-catenin signaling<br>• Promotes proliferation via PI3K/AKT<br>• Increases VEGF-A production<br>• Induces oxidative stress and ER stress	Proliferation, angiogenesis, inflammation
